# Synthesis, Internalization and Visualization of *N*-(4-Carbomethoxy) Pyrrolidone Terminated PAMAM [G5:G3-TREN] Tecto(dendrimers) in Mammalian Cells †

**DOI:** 10.3390/molecules25194406

**Published:** 2020-09-25

**Authors:** Maciej Studzian, Paula Działak, Łukasz Pułaski, David M. Hedstrand, Donald A. Tomalia, Barbara Klajnert-Maculewicz

**Affiliations:** 1 Department of General Biophysics, Faculty of Biology and Environmental Protection, University of Lodz, 141/143 Pomorska St., 90-236 Lodz, Poland; maciej.studzian@biol.uni.lodz.pl (M.S.); paula.dzialak@o2.pl (P.D.); 2 Department of Molecular Biophysics, Faculty of Biology and Environmental Protection, University of Lodz, Banacha 12/16, 90-237 Lodz, Poland; lukasz.pulaski@uni.lodz.pl; 3 Laboratory of Transcriptional Regulation, Institute of Medical Biology PAS, Lodowa 106, 93-232 Lodz, Poland; 4 National Dendrimer & Nanotechnology Center, NanoSynthons LCC, 1200 N. Fancher Avenue, Mt. Pleasant, MI 48858, USA; david.hedstrand@nanosynthons.com; 5 Department of Chemistry, University of Pennsylvania, Philadelphia, PA 19104, USA; 6 Department of Physics, Virginia Commonwealth University, Richmond, VA 23173, USA; 7 Leibniz Institute of Polymer Research, 01397 Dresden, Germany

**Keywords:** tecto(dendrimer), PAMAM, NTIL, blue fluorescence, pyrrolidone, transfection

## Abstract

Tecto(dendrimers) are well-defined, dendrimer cluster type covalent structures. In this article, we present the synthesis of such a PAMAM [G5:G3-(TREN)]-*N*-(4-carbomethoxy) pyrrolidone terminated tecto(dendrimer). This tecto(dendrimer) exhibits nontraditional intrinsic luminescence (NTIL; excitation 376 nm; emission 455 nm) that has been attributed to three fluorescent components characterized by different fluorescence lifetimes. Furthermore, it has been shown that this PAMAM [G5:G3-(TREN)]-*N*-(4-carbomethoxy) pyrrolidone terminated tecto(dendrimer) is able to form a polyplex with double stranded DNA, and is nontoxic for HeLa and HMEC-1 cells up to a concentration of 10 mg/mL, even though it accumulates in endosomal compartments as demonstrated by its unique NTIL emission properties. Many of the above features would portend the proposed use of this tecto(dendrimer) as an efficient transfection agent. Quite surprisingly, transfection activity could not be demonstrated in HeLa cells, and the possible reasons are discussed in the article.

## 1. Introduction

### 1.1. Historical Overview

Over the past four decades, dendrimers and dendritic polymers have created international interest among a diverse range of disciplines and researchers. During the first decade (i.e., 1980s), after the polyamidoamine (PAMAM) dendrimer discovery [1], there arose some skepticism over theoretical claims that these structures could be synthesized as truly monodispersed products that amplified according to a mathematically predictable model at each generation. As such, there was measurable resistance to this new concept and fewer than a dozen peer reviewed dendrimer papers appeared in the literature during that first decade. At that time, many traditional polymer chemists believed that discrete, monodispersed macromolecular structures (i.e., proteins, DNA/RNA, etc.) were attainable only via biological processes, and similar well-defined, abiotic macromolecular analogues were impossible to produce by synthetic methods. Fortunately, this perspective began to change dramatically during the early 1990s [2]. It was at that time that analytical and protein scientists developed mass spectrometry equipment/protocols suitable for characterizing masses of high molecular weight proteins. These new methodologies were quickly applied to the determination of higher molecular weight PAMAM dendrimer masses at the Dow Chemical Co. [3]. Consistent with the original concept [1], these new mass spectrometry protocols demonstrated unequivocally that dendrimers could be synthesized in generational sequences to yield mathematically predictable, monodispersed macromolecular structures [4]. Historically speaking, dendrimers not only became recognized as first examples of a 4th major new architectural polymer class, but they were also distinguished from traditional polymers by their monodisperse/nanoscale sizes which could be systematically controlled by simply advancing the generation level [2,5]. Furthermore, it was shown that the chemical compositions of the three key architectural components of dendrimers, namely (1) cores, (2) interior branches, as well as (3) surface chemistries, could be modified in nearly an unlimited fashion to create vast libraries of discrete, soft nanoparticles with diverse physicochemical properties and a broad range of interior elemental compositions.

It was at this point in the early history of dendrimers that Profs. J.-P. Majoral and A.-M. Caminade (i.e., Toulouse, France) reported the first seminal examples of dendrimers containing a main group elements such as phosphorus [6]. Since that time, this French team has been widely recognized for contributing some of the most innovative concepts/products in the field as they have remained one of the top three most prolific groups based on total dendrimer publications [5]. The dendrimer world has indeed been substantially enriched by these many seminal contributions. Thanks to this early pioneering work, dendrimers, dendrons, dendritic/hyperbranched polymers are now widely recognized with >62,000 patent/literature citations (Web of Science; 21 August 2020), wherein these discrete, well-defined soft nanoparticles are broadly utilized throughout the burgeoning field of nanotechnology (i.e., soft superatoms) [7] and the biomedical sciences [8].

### 1.2. Dendrimer Properties of Value in Drug Delivery and Nanomedicine

One of the first synthesized and hence most studied dendrimer families is the poly(amidoamine) (PAMAM) dendrimers. It is now widely recognized that these Tomalia type PAMAM and other dendrimers possessing interiors derived from “symmetrical branch cells” manifest unique interior void spaces/cavities suitable for hosting guest molecules [9]. In contrast, Denkewalter poly(l-lysine) (PL) dendrimers, which contain “asymmetrical branch cells” do not exhibit interior void space for hosting guest molecules. As such, symmetrical branch cell dendrimers have been used extensively as “unimolecular micelles” for the encapsulation and delivery of a wide range of drugs and active pharmaceutical ingredients [10]. Furthermore, many poly(valent) dendrimer surface moieties (i.e., amino or carboxylic, etc. moieties) have been exploited to bind various molecules/drugs/particles of interest either by direct covalent conjugation or supramolecular exo-complexation. These features make PAMAM dendrimers excellent candidates as drug carriers/encapsulators, imaging agents, gene carriers [11,12,13,14] or as actual active drugs/pharmaceutical agents [15,16].

A critical requirement for using any dendritic structures in drug delivery systems is that they exhibit biocompatibility, do not adversely stimulate/suppress the immune system nor show unacceptable cytotoxic effects [17,18]. The most widely recognized source of dendrimer toxicity is generally associated with cationic surface charge and has been comprehensively demonstrated in the case of amine terminated PAMAM dendrimers. In order to enhance biocompatibility, various surface modifications have been applied [19], wherein surface glycosylation or PEGylation are most commonly used to improve these properties; however, neither of these options is perfect. For example, glycosylation may create both anti-inflammatory and proinflammatory properties [20,21]. On the other hand, PEGylation has been shown to undergo radical based oxidation to produce fragmentation products known to elicit immune responses [22]. As a consequence, there is currently an active quest for PEGylation alternatives in the drug delivery area. That withstanding, such a PEGylation alternative has recently been reported by Tomalia et al. [23]. This alternative involves facile conversion of PAMAM dendrimer surface amines to *N*-(4-carbomethoxy) pyrrolidone moieties which appear to manifest very promising biocompatibility features [24]. It was shown that such a modification dramatically inhibits activation of proinflammatory signals in human monocytes [17] as well as substantially reducing cytotoxicity [25,26]. By analogy to the term PEGylation, these transformations have been referred to as “pyrrolidonylations” [23] and have been proposed as an alternative protocol to PEGylation for cloaking either dendrimer carriers, proteins or drugs. The objective of this cloaking process is to render drugs/drug carriers stealthy to in vivo protein opsonization, thereby reducing complement activation properties, while enhancing drug circulation times and reducing cytotoxicity. Furthermore, it should be noted that dendrimer surface “pyrrolidonylation” substantially enhances so-called “non-traditional intrinsic luminescence”(NTIL) properties [23,27,28]. This fluorescence enhancement allows direct in vivo bioimaging of the dendrimer nanoparticles without any need for conjugating traditional dyes or external staining. These studies demonstrated that pyrrolidone modified PAMAM-dendrimers manifest dramatically enhanced emission intensities (i.e., >50 fold) compared to unmodified amine terminated PAMAM dendrimers [28,29]. Recent proposed mechanisms to account for the NTIL phenomenon have concluded that these inexplicable blue emissions are largely due to the aggregation/clustering and/or physico-chemical confinement of normally non-emissive, electron-rich, hetero-atomic, functionalized moieties, (HASLs) (i.e., heteroatomic subluminophores) [30]. As such, the architectural immobilization of certain HASLs within the interior of PAMAM dendrimers led to some of the first reported examples of NTIL in the literature [31,32]. A logical extension of these NTIL mechanism principles suggests that covalent aggregates of PAMAM dendrimers (i.e., megamers or core-shell tecto(dendrimers)) might be expected to produce prototypes exhibiting substantially enhanced NTIL properties. In order to test this premise, a highly congested tecto(dendrimer) prototype was conceived and synthesized for evaluation as an in vivo NTIL imaging agent in biological cells as well as for its potential as a drug delivery vector.

### 1.3. Core-Shell Tecto(dendrimers); Synthesis and Property Evaluation for Life Science Applications

Within the context of architectural types, core-shell tecto(dendrimers) are well-defined dendri-clusters/assemblies and constitute a subset of poly(dendrimers) referred to as “megamers” [33,34,35]. The first examples of core-shell tecto(dendrimers) were reported by the Tomalia group in 1994 and 2002; however, they have received relatively little attention until the present. The first synthesis of so-called “shell saturated” core-shell tecto(dendrimers) was published in 2000 [36]. In this case, a limited amount of amine terminated PAMAM dendrimer (i.e., referred to as the core reagent) was combined with a large excess of carboxylic acid terminated PAMAM dendrimer, (i.e., referred to as the shell reagent) in the presence of LiCl. This combination yielded a charge neutralized core reagent dendrimer that was completely surrounded (i.e., surface saturated) by shell reagent dendrimer to produce a highly organized, core-shell type supramolecular structure. This core-shell supramolecular assembly was then covalently fixed by adding a carbodiimide coupling reagent to produce amide linkages between the contacting core and shell components. This general protocol produced an ordered subset of megamers referred to as shell saturated, core-shell tecto(dendrimers). This tecto(dendrimer) category was coined to designate the fact that essentially all available surface area on the core dendrimer reagent was systematically and totally occupied by dendrimer shell components. This was possible due to charge neutralization interactions between core and shell components that could be annealed to provide the most efficient parking order for the shell components before covalently fixing core-shell surface positions with amide forming carbodiimide reagents.

**Figure 1:**
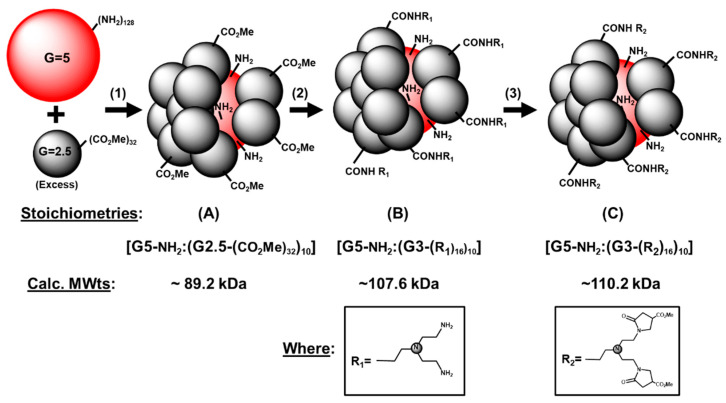
Reaction Conditions: Structure (**A**); Step (1), MeOH, 25× equivalent excess of [DAB:core] dendri-[poly(amidoamine)]-(CO_2_Me)_32_, (G2.5) (DAB-1,4-diaminobutane) with [DAB:core] dendri-[poly(amidoamine)]-(NH_2_)_128_ (G5), (25 days/40 °C); Structure (**B**); Step (2), MeOH, 100× equivalent excess of tris-[2-(aminoethyl)amine], (3 days/4 °C; 8 h/25 °C); Structure (**C**); Step (3), MeOH, dimethyl itaconate (10% excess/amino group), (12 h/25 °C). Where: R_1_ = tris-*N*,*N*′,*N*″[2-(aminoethyl)amine] (TREN) and R_2_ = *N*-(4-carbomethoxy) pyrrolidone terminated TREN moieties.

Subsequently, a second semiordered category of megamers referred to as partial shell filled, core-shell tecto(dendrimers) was reported in 2002 [37]. This class of core-shell tecto(dendrimers) is defined by less ordered, more random core-shell attachment sites. This category is synthesized by a direct reaction of reactive core-shell components to produce covalent linkages. More specifically, this protocol does not involve the formation of a charge neutralized, core-shell supramolecular assembly that may be annealed to establish ordered and complete saturation of the core surface prior to covalent core and shell conjugation. As such, it is differentiated from the “saturated shell” type tecto(dendrimer) category by directly forming random covalent linkages between the core and shell components until all accessible reactive core surface space is filled. Due to these random, unordered covalent binding events, the core reagent surface area is less efficiently filled by the shell reagent dendrimers. In fact, only 40–66% shell saturation is observed [37] relative to total shell saturation levels that may be mathematically predicted by the Mansfield-Tomalia-Rakesh equation [38]. As a consequence, well-defined domains of either nucleophilic/electrophilic nanoclefts or nanocusps are created due to the random, unordered conjugation of shell components to the core. In this present work, nucleophilic clefts (i.e., occupied by primary-NH_2_) are expected to result by attaching electrophilic (i.e., ester terminated) dendrimer shell components to a nucleophilic (i.e., amine terminated) dendrimer core component, as described in Figure 1. A comprehensive review of these superstructured poly(amidoamine) dendrimer based nanoconstructs and their applications in nanomedicine has been recently reported by [39].

Dendrimers exhibit numerous properties which distinguish them as excellent candidates for drug delivery agents: it is most notable that they are well-defined nanoparticles possessing modular, modifiable structures [40]. As such, they can be fine-tuned both with regard to interactions with potential cargo (complex formation, encapsulation, conjugation) and with target cells (nontoxicity, bio-orthogonality, internalization mechanism and kinetics, intracellular fate) [41,42,43,44]. Since unmodified amine-terminated PAMAM dendrimers are largely suboptimal for many direct biological applications due to their toxicity, there is growing attention focused on the engineering of their “critical nanoscale design parameters” (CNDPs) in order to enhance certain properties [25,45,46,47]. As part of this quest, we turned our attention to tecto(dendrimers), a ternary conjugate of one large (core) dendrimer with a number of smaller ones at the periphery (shell) [36,37,48]. We decided to combine this approach with surface modification by pyrrolidone moieties, since they are known to confer biotolerance and enhance nontraditional intrinsic luminescence (NTIL) with the potential for direct bioimaging [24,26,29].

## 2. Results

### 2.1. Synthesis

In this present work, a core-shell tecto(dendrimer) series bearing nucleophilic nanoclefts was prepared by allowing a limited amount of nucleophilic dendrimer core reagent (i.e.,-NH_2_) to react with an excess of electrophilic functionalized dendrimer shell reagent (i.e.,-CO_2_Me) to produce an amide linked partial shell filled, core-shell tecto(dendrimer)*,* bearing nucleophilic clefts (i.e., primary-NH_2_) and electrophilic cusps (i.e.,-CO_2_Me), (Figure 1). In our earlier work, we observed 53–66% shell filling leading to the synthesis of Structure (A). This represents the attachment of 8–10 shell components (i.e., PAMAM G2.5 shell attachment to a PAMAM G5 core) out of the theoretical prediction of 15 shell components according to the Mansfield-Tomalia-Rakesh equation [38]. In this present work, we observed the attachment of 10 out of 15 shell components according to electrophoretic (polyacrylamide gel electrophoresis; PAGE) analyses, thus allowing us to propose stoichiometries and calculate estimated molecular weights for Structures (A)–(C) as described in Figure 1.

The new “partial shell filled” PAMAM [G5:G3-TREN]-*N*-(4-carbomethoxy) pyrrolidone terminated core-shell (tecto)dendrimers were synthesized by slightly modifying our earlier published procedures [36,37]. This minor modification involved using tris-*N*,*N*′,*N*″[2-(aminoethyl)amine] (TREN) versus ethylene diamine to advance Structure (A), (G2.5) ester terminated core-shell tecto(dendrimer) to the next generation level (i.e., G3-TREN, amine terminated),Structure (B). Since the tris-*N*,*N*′,*N*″[2-(aminoethyl)amine] (TREN) reagent contains a branch juncture, this modification produces an additional terminal group amplification by doubling the primary amine groups on the surface of Structure (B). More specifically, this modification involved the following three steps:

Step (1) Reaction of a limited amount of [DAB:core] dendri-poly(amidoamine)-(NH_2_)_128_,(G5);(PAMAM) dendrimer with an excess (i.e., ×25 equivalents) of [DAB:core] dendri-poly(amidoamine)-(-CO_2_Me)_32_,(G2.5), (PAMAM) dendrimer in the presence of LiCl for 25 days/40 °C) produced PAMAM [core(G5):shell(G2.5)]-CO_2_Me terminated tecto(dendrimer) Structure (A). In our previous work, we observed shell saturation levels for the core-shell Structure (A) that varied between 53 and 66% (i.e., 8–10 shell tectons) versus a theoretical prediction of 15 shell tectons for a 100% saturation level as predicted by the Mansfield-Tomalia-Rakesh equation [36,38]. In this present work, Structure (A) appeared to possess ~9–10 dendrimeric shell tectons as determined by comparison to monomeric PAMAM G6 (MWt = 58.0 kDa) and PAMAM G7 (MWt = 116.5 kDa), respectively, using PAGE analyses (Supplementary Material, Figure S3). Excess dendrimer shell reagent G2.5 was removed by ultrafiltration to yield Structure (A). Note, it is very important not to isolate Structure (A) in a neat form. This product must be handled as a solution or rapid oligomerization, including cross-linking, which may occur due to amidation reactions involving nanocleft amino moieties and nanocusp ester groups.

Step (2) After removing excess shell reagent, G2.5, the ester terminated core-shell tecto(dendrimer), Structure (A) was allowed to react with a large excess (×100 equivalent) of tris-[2-(aminoethyl) amine], (TREN), to yield PAMAM core(G5)-(NH_2_):shell(G3)-(TREN-NH_2_) terminated tecto(dendrimer)**,** Structure (B). This product could be isolated as a white solid by ultrafiltration or dialysis.

Step (3) Allowing Structure (B) to react with a slight excess (i.e., 10%) of dimethyl itaconate converted all accessible primary amines to *N*-(4-carbomethoxy) pyrrolidone moieties to yield the final PAMAM [core (G5):shell (G3)-(TREN)]-*N*-(4-carbomethoxy) pyrrolidone terminated tecto(dendrimer), Structure (C)**.** This final modified product was obtained as a white solid after ultrafiltration. It differs from all previously reported core-shell tecto(dendrimers), wherein the branch cells involved in the formation of the final (G3) shell layer are derived from tris-[2-(aminoethyl)amine], (TREN). This product was characterized by FTIR, ^1^H/C^13^-NMR and electrophoresis (PAGE). A comparison with standard protein markers, other authentic relevant core-shell tecto((dendrimers) and appropriate *N*-(4-carbomethoxy) pyrrolidone terminated PAMAM monomeric dendrimers corroborates the proposed Structure (C), which appears to be present as a mixture of monomeric, dimeric and trimeric species as determined by PAGE analyses (Supplementary Material, Figure S4). A comparison with standard protein markers revealed three discrete electrophoretic bands with estimated molecular weights of ~115, ~225 and ~325 kDa, respectively. These values correspond to molecular weights expected for monomeric, dimeric and trimeric forms, respectively, for core-shell tecto(dendrimer), Structure (C).

A survey of the literature reveals that fewer than a handful of core-shell (tecto)dendrimers have been reported to date [37,49,50]. However, core-shell (tecto)dendrimers have been examined as efficient, multivalent nanoscale platforms for the delivery of pharmaceuticals [51,52]. Due to their dendrimeric multiplicities and larger aggregate dimensions, they possess substantially more surface groups and internal cavity space. This enhanced interior void space endows them with variable sizes and well-defined chemistry, which could enable the transfer of diverse or multiple drug combinations [50]. Quite surprisingly, it has been shown that certain PAMAM core-shell tecto(dendrimers) possess intrinsic antitumor activity, wherein they exhibit selective cytotoxicity to melanoma cells but do not affect healthy cells [53].

In summary, activity in this area is in its infancy, and there remains a need for the synthesis and development of new core-shell tecto(dendrimer) structures. It is hoped these tecto(dendrimer) structures may combine desirable features such as: high biocompatibility, higher multiplicity, polyvalent features (i.e., external binding/interior encapsulation sites) as well as enhanced intrinsic fluorescence properties (i.e., NTIL emissions) suitable for in vivo bioimaging in combination with various drug delivery applications [30].

### 2.2. Selected Physicochemical Properties of PAMAM [G5:G3-(TREN)-N-(4-carbomethoxy) pyrrolidone]_10_ Terminated Tecto(dendrimer)

In order to determine the level of accessible amine functionalized nanoclefts present within the structure of PAMAM [G5:G3-(TREN)-*N*-(4-carbomethoxy) pyrrolidone]_10_ terminated tecto (dendrimer), Structure (C), we utilized a quantitative ninhydrin assay. As a positive control, we used amino-terminated PAMAM G5 dendrimer, wherein its surface presents theoretical 128 primary amino groups. Ninhydrin showed a positive reaction with PAMAM [G5:G3-(TREN)]-*N*-(4-carbomethoxy) pyrrolidone terminated tecto(dendrimer) (C), thus confirming the presence of residual primary amino groups on this nanoparticle. A quantitative ninhydrin analysis/comparison indicated there was ca. 84-times less Ruhemann’s purple produced in the reaction with PAMAM [G5:G3-(TREN)]-*N*-(4-carbomethoxy) pyrrolidone terminated tecto(dendrimer) (C) (i.e., 0.28 absorbance units per mg of the dendrimer) compared with PAMAM G5 amine terminated dendrimer (i.e., 23.92 absorbance units per mg of the dendrimer). This result was further corroborated by the measurement of the electrokinetic potential of PAMAM [G5:G3-(TREN)]-*N*-(4-carbomethoxy) pyrrolidone terminated tecto(dendrimer) (C) solution at pH 7.4 (in HEPES buffer). The average value of zeta potential was equal to +13.425 ± 0.459 mV, confirming the positive charge of the nanoparticle surface most probably arising from protonated primary amino groups presumably residing in the nanoclefts.

**Figure 2:**
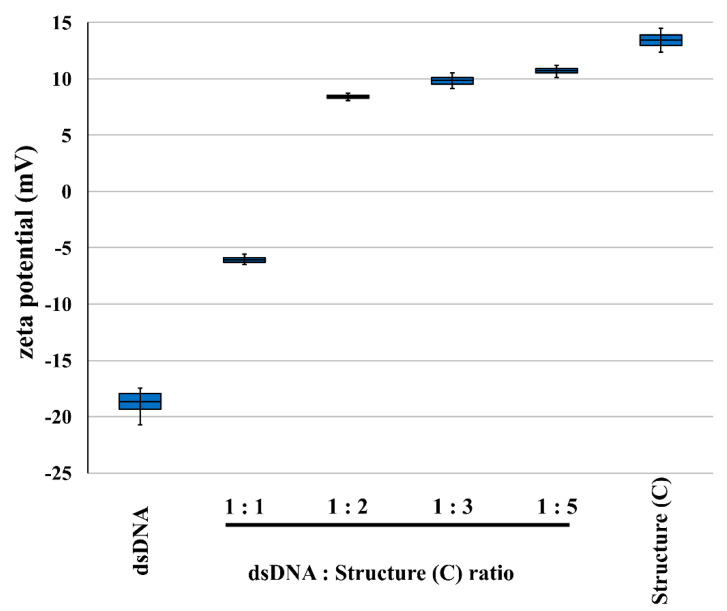
Zeta potentials of dsDNA and PAMAM [G5:G3-(TREN)]-*N*-(4-carbomethoxy) pyrrolidone terminated tecto(dendrimer) (Structure (C)) solutions and their respective dsDNA: PAMAM [G5:G3-(TREN)]-*N*-(4-carbomethoxy) pyrrolidone terminated tecto(dendrimer) (Structure (C)) polyplexes formed at different DNA to dendrimer weight ratios. All data were measured at 25 °C. Error bars represent minimal and maximal values; boxes represent standard error of mean (*n* = 4).

Additionally, we measured changes in zeta potential of the solution of double stranded DNA (plasmid, dsDNA) upon titration with PAMAM [G5:G3-(TREN)]-*N*-(4-carbomethoxy) pyrrolidone terminated tecto(dendrimer) (C). The results are summarized in Figure 2.

In fact, PAMAM [G5:G3-(TREN)]-*N*-(4-carbomethoxy) pyrrolidone terminated tecto(dendrimer) (C) was able to form a polyplex with double stranded DNA and convert its initially strongly negative zeta potentials to positive values with only two times excess ratio (weight).

**Figure 3:**
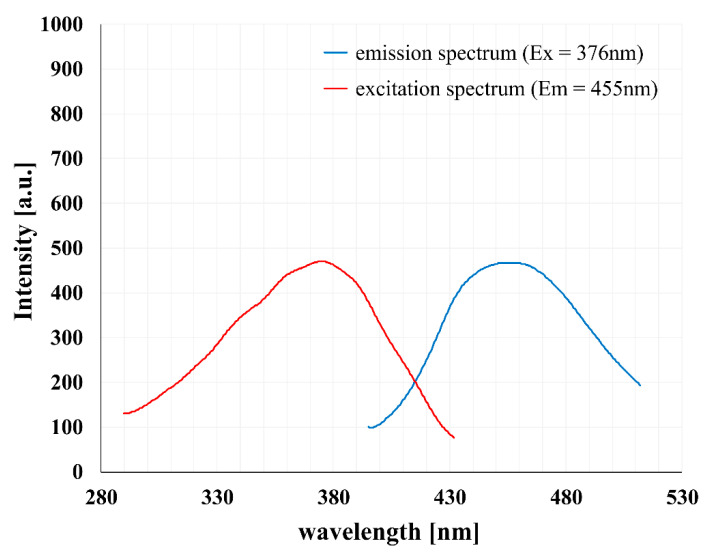
Excitation and emission fluorescence spectra of 1 mg/mL PAMAM [G5:G3-(TREN)]-*N*-(4-carbomethoxy) pyrrolidone terminated tecto(dendrimer), Structure (C) in PBS.

Next, we examined the NTIL type fluorescent properties of PAMAM [G5:G3-(TREN)]-*N*-(4-carbomethoxy) pyrrolidone terminated tecto(dendrimer) (C). Excitation and emission spectra were recorded for 1 mg/mL solution of the polymer in PBS. Measured fluorescence intensities were found to be the same order of magnitude as those recorded for monomeric *N*-(4-carbomethoxy) pyrrolidone (4-CMP) modified PAMAM dendrimers (Figure 3).

Similarly, the values of three fluorescence lifetime components of the fluorescence decay curves measured for intrinsic fluorescence of PAMAM [G5:G3-(TREN)]-*N*-(4-carbomethoxy) pyrrolidone terminated tecto(dendrimer) (C) by the TCSCP method (Table 1) were close to the values obtained for *N*-(4-CMP) PAMAM dendrimers ($\tau_{1}$ ≈ 0.4 ns, $\tau_{2}$ ≈ 3 ns, $\tau_{3}$ ≈ 8 ns) [28]. However, intensity fractions of the respective components were somewhat different (ca. 40% for component 2 in tecto(dendrimer) relative to ca. 10% in *N*-(4-CMP) PAMAM dendrimers, and ca. 50% for component 3 in tecto(dendrimer) relative to ca. 90% in *N*-(4-CMP) PAMAM dendrimers).

### 2.3. Cytotoxicity of PAMAM [G5:G3-(TREN)]-N-(4-carbomethoxy) Pyrrolidone Terminated Tecto(dendrimer) (C)

**Figure 4:**
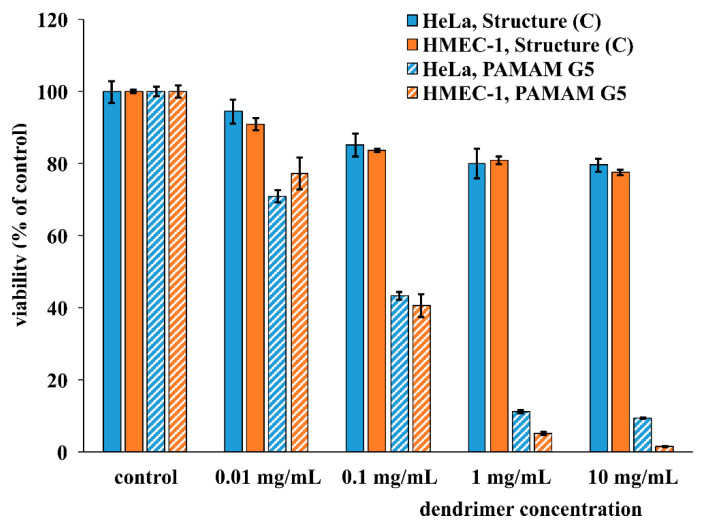
Cytotoxic effects of PAMAM [G5:G3-(TREN)]-*N*-(4-carbomethoxy) pyrrolidone terminated tecto(dendrimer) (C) and PAMAM (G5) dendrimer on HeLa and HMEC-1 cells. Cell viability was determined by the resazurin assay after 24 h treatment with dendrimers. Data are presented as percentage of viability of control (untreated) cells. Error bars represent the standard error (*n* = 10).

Next, we evaluated the cytotoxicity of PAMAM [G5:G3-(TREN)]-*N*-(4-carbomethoxy) pyrrolidone terminated tecto(dendrimer) (C) and amino-terminated PAMAM G5 dendrimer. Resazurin cytotoxicity assay was performed on two distinct mammalian cell lines: HeLa cells (cervical cancer transformed cell line) and HMEC-1 cells (immortalized human microvascular endothelial cells). Prior to the cytotoxicity assay, cells were incubated with nanoparticles in respective fully supplemented growth media for 24 h. Obtained results are summarized in Figure 4. Surprisingly, despite having a positive zeta potential and exhibiting ninhydrin-reactive, free amine groups, PAMAM [G5:G3-(TREN)]-*N*-(4-carbomethoxy) pyrrolidone terminated tecto(dendrimer) (C) was only marginally cytotoxic for HeLa and HMEC-1 cells at the highest tested concentrations (viability ca. 80% for 10 mg/mL concentration in contrast to monomeric, amino-terminated, G5; PAMAM dendrimer (viability less than 10% for 10 mg/mL concentration).

### 2.4. Internalization of PAMAM [G5:G3-(TREN)]-N-(4-carbomethoxy) Pyrrolidone Terminated Tecto(dendrimer) (C) into HeLa Cells

**Figure 5:**
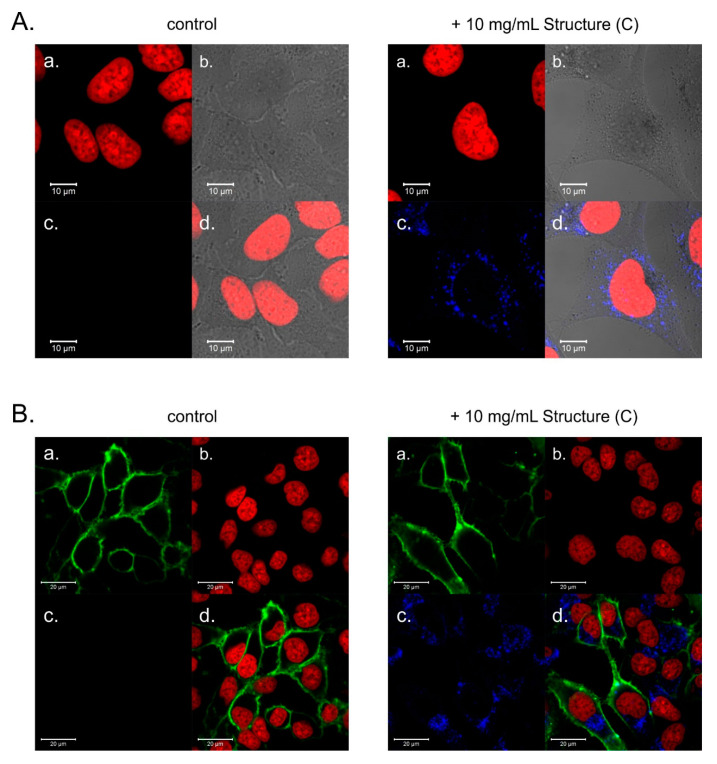
Representative confocal images of untreated (control) and incubated for 24 h with PAMAM [G5:G3-(TREN)]-*N*-(4-carbomethoxy) pyrrolidone terminated tecto(dendrimer) (C) with HeLa cells. (**A**)**.** Cell nuclei were stained with RedDot1. Split images represent: a. cell nuclei, b. transmitted light image, c. blue fluorescence channel (intrinsic fluorescence of PAMAM [G5:G3-(TREN)]-*N*-(4-carbomethoxy) pyrrolidone terminated tecto(dendrimer) (C), d. merge of a., b. and c. (**B**). Cell nuclei were stained with RedDot1; plasma membranes were stained with NeuroDiO. Split images represent: a. plasma membrane, b. cell nuclei, c. blue fluorescence channel (intrinsic fluorescence of PAMAM [G5:G3-(TREN)]-*N*-(4-carbomethoxy) pyrrolidone terminated tecto(dendrimer) (C), d. merge of a., b. and c.

Considering that PAMAM [G5:G3-(TREN)]-*N*-(4-carbomethoxy) pyrrolidone terminated tecto(dendrimer) (C) exhibits significant intrinsic fluorescence and low toxicity, we set out to visualize its potential for internalization into human cells. For this purpose, HeLa cells were incubated with PAMAM [G5:G3-(TREN)]-*N*-(4-carbomethoxy) pyrrolidone terminated tecto(dendrimer) (C) at 10 mg/mL concentration for 24 h. Dendrimer-treated and untreated (control) cells were subsequently stained to fluorescently label cell nuclei and plasma membranes. Representative confocal images are presented in Figure 5. In cells incubated with PAMAM [G5:G3-(TREN)]-*N*-(4-carbomethoxy) pyrrolidone terminated tecto(dendrimer) (C), strong blue intracellular fluorescence corresponding to dendrimer fluorescence emission maximum (emission channel 410–480 nm) can be observed. After 24 h incubation time, tecto(dendrimer) accumulated within HeLa cells in diffused, endosomal compartments.

### 2.5. Potential of PAMAM [G5:G3-(TREN)]-N-(4-carbomethoxy) Pyrrolidone Terminated Tecto(dendrimer) (C) for DNA Transfection into HeLa Cells

**Figure 6:**
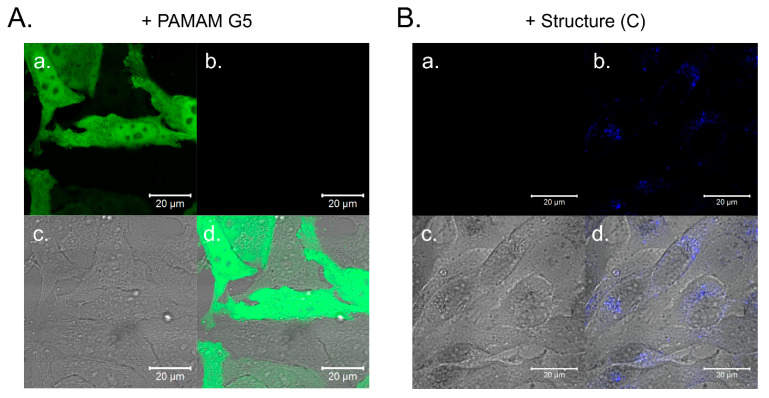
Representative confocal images of HeLa cells transfected using PAMAM G5 (**A**) and PAMAM [G5:G3-(TREN)]-*N*-(4-carbomethoxy) pyrrolidone terminated tecto(dendrimer) (Structure (C)) (**B**). Dendrimers were complexed at 8:1 N/P ratio with expression plasmid encoding TagGFP2, and cells were incubated with this complex for 24 h. Split images represent: a. TagGFP2 fluorescence (green channel), b. nontraditional intrinsic luminescence of the dendrimer (NTIL) (blue channel), c. transmitted light image, d. merge of a., b. and c.

Since we showed that PAMAM [G5:G3-(TREN)]-*N*-(4-carbomethoxy) pyrrolidone terminated tecto(dendrimer) (C) has DNA binding capability and it is considerably less toxic than PAMAM G5 dendrimer (which has previously been demonstrated to be suitable as a transfection agent), we set out to verify the potential usefulness of PAMAM [G5:G3-(TREN)]-*N*-(4-carbomethoxy) pyrrolidone terminated tecto(dendrimer) (C) as a vector for DNA transfection. While we were able to demonstrate that a low percentage of HeLa cells was effectively transfected by amino-terminated PAMAM G5—plasmid DNA polyplex (i.e., expression of a green fluorescent protein—Figure 6A), no transfected cells were observed after incubation with the PAMAM [G5:G3-(TREN)]-*N*-(4-carbomethoxy) pyrrolidone terminated tecto(dendrimer) (C)—plasmid DNA polyplex even after numerous attempts at various DNA concentrations and N/P ratios. However, as shown in Figure 6B, Structure (C): plasmid DNA polyplex was effectively internalized by the cells and was visualized thanks to its characteristic fluorescence.

## 3. Discussion

We characterized the basic physicochemical properties (i.e., relevant to our biological goals) of the novel pyrrolidone-modified tecto(dendrimers) and found them to be consistent with expectations and needs for further application development. Thanks to their special core-shell aggregate architecture, they display a combination of advantageous traits observed for both nonmodified (i.e., amine terminated) and pyrrolidone modified (i.e., pyrrolidonylated) monomeric PAMAM dendrimers. The core-shell structures possess accessible nanoclefts bearing free primary amino groups, which appear to confer cationic surface charge in neutral aqueous solution. However, this cationic property is significantly weaker than in the case of basic unmodified, amine terminated PAMAM. On the other hand, pyrrolidonylation (i.e., 4-CMP modification) of the core-shell tecto(dendrimer) structure confers strongly enhanced NTIL emission features compared to the pyrrolidone modified monomeric PAMAM dendrimers. More specifically, fluorescence lifetime analysis of core-shell tecto(dendrimer) structures confirmed that they manifested relevant NTIL fluorophore features that behaved analogously to previously characterized monomeric, *N*-(4-carbomethoxy pyrrolidone) (4-CMP) terminated PAMAM dendrimers [29]. For example, two relatively strong but comparable fluorophore centers, (i.e., corresponding to fluorescence decay curve components 2 and 3 in this study) are distinctly noted in this analysis [28]. It has previously been demonstrated that the brightest fluorophore (component 3, in our tecto(dendrimers) contributing half of total fluorescence output) is derived from interactions of tertiary amino groups and imidic acid moieties along the terminal PAMAM branches and that its brightness and quantum yield are enhanced by branch stabilization [54,55,56]. In the case of tecto(dendrimers), this stabilization is probably conferred both by the 4-CMP modification as well as by the enhanced rigidity of the core-shell tecto(dendrimer) architecture. This last feature is consistent with a recent NTIL phenomenon mechanism proposed by us [30], which invokes immobilization due to rigidity as a significant parameter for enhancing NTIL emission properties.

Since the pyrrolidone-modified core-shell Structure (C) appeared to be fulfilling certain predicted needs as a potential nanoscale vector for drug delivery in mammalian cells, we next performed a series of critical biochemical/cellular measurements to verify these desirable properties. Firstly, Structure (C) exhibited extremely low toxicity properties when exposed to relatively delicate cells (i.e., derived from vascular endothelium) even at concentrations significantly higher than normally used for practical applications. This finding was surprising and exceeded all expectations. This was especially true in view of its PAMAM chemical structure as well as its larger nanoscale dimensions. Earlier work verified that dendrimers bearing cationic primary amino groups, especially when presented on large, higher generation PAMAM dendrimers, were very deleterious to biocompatibility and generally manifested strong toxicity properties [57]. On the other hand, this result corroborates the basic ideas behind the structural design of PAMAM [G5:G3-(TREN)]-*N*-(4-carbomethoxy) pyrrolidone terminated tecto(dendrimer) (C). As expected, 4-CMP modification successfully masks the external (G3-linked) primary amine groups, which are directly involved in cell membrane/surface receptors interactions that lead to toxicity. On the other hand, internal nanocleft (G5-linked) primary amine groups appear to remain available as cationic sites for charge conferral and biomolecular cargo interactions without exhibiting any toxicity features. Furthermore, these observations may also confirm a broadly held tenet that pyrrolidone-modified molecules (including biomaterials) usually have unexpectedly good biocompatibility properties compared to their basal forms due to the bio-orthogonality of the pyrrolidone moiety [58].

Since nanoparticle based drug delivery applications require a complex sequence of biochemical interactions with the cargo as well as the cell surface to fulfill their role [59], we decided to perform certain mechanistic evaluations before embarking on a phenomenological experiment. As such, we examined the capacity of PAMAM [G5:G3-(TREN)]-*N*-(4-carbomethoxy) pyrrolidone terminated tecto(dendrimer) (C) for binding with DNA by observing changes in surface charge distribution around the macromolecule using zeta potential measurements. This allowed us to not only to confirm the efficacy of DNA polyplex formation, driven by the nanocleft based, cationic amino group charge of the tecto(dendrimer), but also to monitor its stoichiometry with DNA. Considering that plasmid DNA is a very large molecular structure, it is significant that the DNA: core-shell tecto(dendrimer) complexation (i.e., at a 1:1 weight ratio) produced a net negative phosphate charge from the DNA. It was determined that the saturation point for neutrality resided around 1:1.5 (DNA: dendrimer weight ratio).

Subsequently, we verified the capability of cells to take up the tecto(dendrimer) nanoparticle. Here, the nontraditional intrinsic luminescence (NTIL) properties of the tecto(dendrimer) are a crucial feature since they allow us to use direct confocal microscopy to confirm the subcellular localization of cell-bound molecules. While NTIL emission intensities for unmodified, amine terminated PAMAM dendrimers are too weak to allow efficient bioimaging against the background of autofluorescent cellular components, we show that PAMAM [G5:G3-(TREN)]-*N*-(4-carbomethoxy) pyrrolidone terminated tecto(dendrimer) (C) produces NTIL emissions that are bright enough to be effectively imaged within cellular structures without significant interference from background fluorescence. Thus, these core-shell tecto(dendrimer) structures join the ranks of very few available nanoparticles manifesting intrinsic fluorescence emissions strong enough for label-free bioimaging. Although inorganic quantum dots represent such an intrinsically fluorescent nanoparticle category, it is also well known that they exhibit very dangerous heavy metal toxicity properties. This is in sharp contrast to these core-shell tecto(dendrimers) which exhibit virtually no cytotoxicity features at the cellular level. Thus, the usual nanoparticle imaging conundrum (i.e., nontoxic nanoparticles require conjugated labeling to be fluorescent, while fluorescent particles need to be coated for biocompatibility) may be avoided. This now appears to be possible with a single, well-defined nanoparticle type such as PAMAM core-shell tecto(dendrimers). Even more importantly, we show that these core-shell tecto(dendrimer) particles are efficiently internalized by mammalian cells and end up concentrated in an endosomal compartment, pointing to endocytosis rather than pinocytosis as the major uptake mechanism. This highly efficient internalization property is quite surprising, considering its relatively large polymeric structure, thus giving high optimism for future applications in drug delivery systems.

Our final phenomenological experiment concerning DNA delivery, as verified by gene transfection assessment, produced a rather surprising result. More specifically, although we were able to observe very efficient internalization of the DNA-tecto(dendrimer) polyplex, we were unable to detect any significant level of DNA transfection activity based on the lack of reporter protein expression from the DNA plasmid. In the case of unmodified, (G5) amine terminated PAMAM-(NH_2_)_128_ dendrimer, which is strongly toxic to cells, some of the surviving cells were indeed transfected, confirming the literature data that these structures are generally suitable for gene transfection [60]. However, it appears that tecto(dendrimer) architecture changes the DNA binding properties in such a manner as to prevent the efficient delivery of the cargo plasmid to the nucleus, where it has to be transported and located for expression. This may be due to one of several reasons: either the interaction between tecto(dendrimer) and DNA is too strong for efficient release within the cell, the dendrimer-DNA complex is trapped within the lysosomal pathway and cannot be released to cytoplasm or the DNA-tecto(dendrimer) polyplex is too large to enter the nucleus, which are all prerequisite steps in the transfection process. It may be possible that the lack of flexibility of the pyrrolidone-decorated tecto(dendrimer) structure, may restrict the endosomal escape of the DNA, which is also crucial for effective transfection activity [61]. Although this assessment does not indicate the PAMAM [G5:G3-(TREN)]-N-(4-carbomethoxy) pyrrolidone terminated tecto(dendrimer) (C) functions in its present form as an effective DNA transfection vector, it does suggest many other possibilities for drug delivery as well as possible structural modifications for enhancing effective DNA transfection, which we intend to explore in the future.

## 4. Materials and Methods

### 4.1. Synthesis of Modified PAMAM G5:G2.5 Tecto (Dendrimer)

All PAMAM dendrimer reagents (i.e., core reagent-[DAB:core]-dendri-{poly(amidoamine)-(NH_2_)_128_}(PAMAM)(G5) and shell reagent-[DAB:core]-dendri-{poly(amidoamine)-(CO_2_Me)_32_} (PAMAM);(G2.5)) were provided by NanoSynthons, LLC, Mt. Pleasant, MI, USA as ultrapure PAMAM**** products. The characterization of monomeric PAMAM dendrimer core reagents and shell reagents, using electrophoretic analyses (PAGE), confirmed appropriate single band PAMAM dendrimer products with low dimer content and no trailing generation side products (i.e., Supplementary Material).

#### 4.1.1. Synthesis of PAMAM, [G5:G2.5-(CO_2_Me)]_10_, Structure (A)

A modified version of a procedure (Route I) reported in (Tomalia et al., 2002) was used as described below:

The “shell reagent dendrimer”, namely; [Core:DAB]:dendri-{PAMAM-(CO_2_Me)_32_};(G = 2.5); (MWt = 6036 Da); (2.0 g, 3.3 × 10^−4^ moles); note, 100× equivalents per PAMAM G5; core reagent) was charged into a 25 mL single necked, round bottomed flask equipped with a magnetic stirrer, containing 5 mL of methanol and 6.0 mg.

An amount of 1.4 × 10^−3^ moles of lithium chloride; note, 4× equivalents of LiCl per mole of PAMAM shell reagent G2.5. To this stirred mixture was added the “core reagent dendrimer”; [Core:DAB]; dendri-{PAMAM}-(NH_2_)_128_}; (G = 5.0); (MWt = 28,854 Da); (95 mg 3.3 × 10^−6^ mol) dissolved in 2 mL MeOH in a dropwise manner over 5 min. while stirring. This reaction mixture was sealed under N_2_ and heated at 40 °C for 25 days. Periodic analysis by SEC revealed the production of the higher molecular weight core-shell tecto(dendrimer) product, which was essentially equivalent in size to a PAMAM (G7) dendrimer. This was further confirmed by PAGE analysis which indicated a molecular weight of ~116 kDa (Supplementary Material, Figure S3). Formation of the desired ester terminated, core-shell (tecto) dendrimer was monitored by ^13^C/^1^H-NMR, SEC, PAGE and FTIR spectroscopy and found to be essentially complete after 25 days based on no further amide formation. This ester terminated core-shell tecto(dendrimer) product could be isolated by ultrafiltration using an Amicon 30 kDa cut-off, regenerated cellulose membrane and after collecting a total of 9 L permeate. Note, this product should either be immediately pacified by treatment with tris(2-hydroxymethyl)aminomethane as described in [37] or used directly for the synthesis of core-shell tecto(dendrimer), Structure (B) as described below.

#### 4.1.2. Synthesis of PAMAM, [G5:G3-(TREN)]_10_, Structure (B)

The crude reaction mixture of Structure (A), including remaining G2.5 ester terminated dendrimer shell reagent, was added to a stirred solution of excess tris-(2-aminoethyl) amine (TREN); (i.e., 100 equivalent excess of TREN relative to the amount of original “ester terminated dendrimer shell reagent” used) in 50 mL methanol. The amidation of all ester functions was monitored by FTIR and found to be complete after 3 days at 4 °C and 8 h at room temperature. The excess TREN was first extracted away with several (10 mL) toluene washings. Complete removal of TREN together with any remaining amine terminated G3, dendrimer shell reagent was accomplished by exhaustive ultrafiltration through an Amicon 30 KDa cut-off, regenerated cellulose membrane and after collecting at least 8 L permeate. This methanol soluble product could be isolated as a white solid after lyophilization and gave the ^13^C-NMR as shown in Supplementary Material Figure S1.

#### 4.1.3. Synthesis of PAMAM, [G5:G3-(TREN)-*N*-(4-Carbomethoxy) Pyrrolidone]_10_, Structure (C)

This white solid, PAMAM, [G5:G3-(TREN)]_10_, Structure (B) reaction product was allowed to react with dimethyl itaconate (i.e., ~10% excess/amino group) in methanol for 12 h at room temperature to give the final PAMAM, [G5:G3-(TREN)-*N*-(4-carbomethoxy) pyrrolidone]_10_ terminated, Structure (C). This methanol soluble product was obtained as a white solid after lyophilization and confirmed by ^13^C-NMR and electrophoretic (PAGE) analyses (Supplementary Material, Figures S2 and S4).

### 4.2. Cell Culture

HeLa and HMEC-1 cell lines were purchased from American Type Culture Collection and maintained under standard conditions. HeLa cells were cultured in DMEM medium with high glucose (4.5 g/L) and 10% fetal bovine serum (FBS). HMEC-1 cells were cultured in MCDB131 medium supplemented with epidermal growth factor (10 ng/mL), hydrocortisone (1 µg/mL), glutamine (10 mM) and 10% FBS. Cells were subcultured three times per week.

### 4.3. Ninhydrin Assay

Dendrimers were dissolved in methanol. An amount of 50 µL of various concentrations of (G5)PAMAM-(NH_2_)_128_ dendrimer (between 0.1 and 1 µg/µL) and PAMAM [G5:G3-(TREN)]-*N*-(4-carbomethoxy) pyrrolidone terminated tecto(dendrimer) (C) (between 10 and 50 µg/µL) were mixed with equal volumes of 96% ethanol and 0.25% ninhydrin solution in n-butanol. Mixtures were incubated at 85 °C for 10 min and cooled to room temperature. The absorbance of the samples was measured at 570 nm and relative quantity of ninhydrin-reactive amino groups was calculated from linear plots of absorbance versus concentration and was given in absorbance units per mg of the nanoparticle.

### 4.4. Physical Characterization of PAMAM [G5:G3-(TREN)]-N-(4-carbomethoxy) pyrrolidone]_10_ Terminated Tecto(dendrimer) Structure (C)

Excitation and emission fluorescence spectra of PAMAM [G5:G3-(TREN)]-*N*-(4-carbomethoxy) pyrrolidone terminated tecto(dendrimer) (C) were recorded at room temperature on a LS55 spectrofluorometer (Perkin-Elmer). Fluorescence lifetimes of nontraditional intrinsic luminescence (NTIL) emissions of PAMAM [G5:G3-(TREN)]-*N*-(4-carbomethoxy) pyrrolidone terminated tecto(dendrimer) (C) were measured in PBS (pH 7.4) as described previously [28]. Zeta potentials were measured in 10 mM HEPES buffer (pH 7.4) with Zetasizer Nano (Malvern Panalytical). For the titration of dsDNA with PAMAM [G5:G3-(TREN)]-*N*-(4-carbomethoxy) pyrrolidone terminated tecto(dendrimer), Structure (C), a 10 µg/mL concentration of pTagGFP2-N (Evrogen) was mixed with an increasing amount of dendrimer added from 10 mg/mL stock solution.

### 4.5. Cytotoxicity Assay

Cells were seeded into 96-well black plates at a density of 2 × 10^4^ cells per well and treated with increasing concentrations of tecto(dendrimer) for 24 h in respective fully supplemented culture media. Following the incubation and single wash with medium, resazurin was added to the culture medium to a final concentration of 10 µg/mL and plates were incubated at 37 °C in darkness to allow conversion of resazurin to resorufin. Resulting fluorescence of metabolized resazurin was measured after 30, 60 and 90 min at 530 nm excitation and 590 nm emission using a microplate reader. Cell viability was derived from the slope of resorufin fluorescence intensity increase in time and was presented as a percentage of untreated control.

### 4.6. Transfection with (G5) Amine Terminated PAMAM Dendrimers

At 24 h prior to transfection experiment, HeLa cells were seeded onto thin-glass bottomed 96-well plate at a density of 2 × 10^4^ per well. Amounts of 1.1, 2.2 or 3.3 µg of pTagGFP2-*N* plasmid encoding green fluorescent protein (TagGFP2) were dissolved in 55 µL Opti-MEM medium (Gibco), to which subsequently (G5):PAMAM-(NH_2_)_128_ dendrimer or PAMAM [G5:G3-(TREN)]-*N*-(4-carbomethoxy) pyrrolidone terminated tecto(dendrimer) (C) was added in various N to P ratios (between 4 to 1 and 80 to 1). The mixture was incubated at room temperature for 15 min, and resulting DNA-dendrimer polyplexes were next added to cell cultures to the final concentration of 0.1 to 0.3 µg DNA per well. After 24 h incubation, cells were visualized by confocal microscopy.

### 4.7. Confocal Microscopy

The aliquots of 2 × 10^4^ of HeLa cells were seeded per well on thin-glass bottomed 96-well plate (SensoPlate, Greiner Bio-One, Kremsmünster, Austria) and were incubated for 24 h under standard conditions in culture medium with or without the presence of PAMAM [G5:G3-(TREN)]-*N*-(4-carbomethoxy) pyrrolidone terminated tecto(dendrimer) (C) added to the final concentration of 10 mg/mL. Subsequently, cells were washed once with Hanks’ Balanced Salt solution (HBSS), and their nuclei were stained for 30 min in room temperature with RedDot1 dye (1:200, Biotium, Hayward, CA, USA). Next, cell plasma membranes were stained for 2 min with NeuroDiO (1:200, Biotium), cells were washed twice with HBSS and analyzed immediately. Confocal images were obtained with an LSM 780 microscope equipped with an LCI Plan-Neofluar 63×/1.3 Imm Corr DIC objective, 405 nm laser diode and In Tune™ tunable excitation laser system (Zeiss). Stained cells were imaged to visualize the intrinsic fluorescence of PAMAM [G5:G3-(TREN)]-*N*-(4-carbomethoxy) pyrrolidone terminated tecto(dendrimer) (C) in the blue channel (excitation 405 nm, emission 410–480 nm), plasma membranes in the green channel (excitation 490 nm, emission 510–580 nm) and nuclei in the far-red channel (excitation 643 nm, emission 660–725 nm). Additionally, transmitted light images were recorded with an external detector (excitation 643 nm).

## 5. Conclusions

In summary, we observed interesting and somewhat unexpected physical/biochemical properties for the pyrrolidone-modified PAMAM [G5:G3-(TREN)]-*N*-(4-carbomethoxy) pyrrolidone terminated tecto(dendrimers), such as Structure (C). This large, somewhat complex synthetic structure exhibits many similarities to biological protein-based complexes, especially its lack of toxicity despite the presence of unique nanocleft based primary amine moiety site. It is important to remember that this is an example of an architecture type which can and will be further developed, with easily modifiable facades as well as internal cavities that can be a platform for interaction with various binding partners. We demonstrate three crucial properties warranting further studies in drug delivery, namely these tecto(dendrimers) exhibit intense nontraditional intrinsic fluorescent (NTIL) emission properties suitable for in vivo applications, they complex DNA efficiently, they enter biological cells readily and they exhibit low cytotoxicity. The complexity and rigidity of the core-shell tecto (dendrimer) structure may be crucial for each of these characteristics and deserves further examination. Although the direct use of these core-shell tecto(dendrimer) structures in their current form failed as vectors for DNA transfection applications, the unique nontoxic cationic character manifested by these structures as well as their highly enhanced NTIL emission properties allows for their use as biocompatible, highly fluorescent, NTIL emissive nanoparticles for direct biological imaging.

## Figures and Tables

**Table 1:** Fluorescence lifetimes (τ) and intensity fractions (f) or respective fluorescence decay curve components of PAMAM [G5:G3-(TREN)]-*N*-(4-carbomethoxy) pyrrolidone terminated tecto(dendrimer) (C) (pH 7.4). Values are shown as average ± s.e.m. (*n* = 4).

	$\tau_{1}$	$f_{1}\;(\%)$	$\tau_{2}$	$f_{2}\;(\%)$	$\tau_{3}$	$f_{3}\;(\%)$
PAMAM [G5G3-(TREN)]-*N*-(4-carbomethoxy) pyrrolidone terminated; tecto(dendrimer)(C)	0.43 ± 0.01	5.86 ± 0.22	2.35 ± 0.03	44.22 ± 0.77	7.11 ± 0.06	49.93 ± 0.98

## Supplementary Materials

The following are available online. Figure S1: ^13^C-NMR spectra of core-shell tecto(dendrimer) Structure (B); Figure S2: ^13^C-NMR spectra of core-shell tecto(dendrimer) Structure (C); Figure S3: PAGE analysis of PAMAM [G5:G2.5-(CO_2_Me)]_10_, Structure (A); Figure S4: PAGE of core-shell PAMAM, [G5:G3-TREN]-*N*-(4-carbomethoxy) pyrrolidone terminated, Structure (C).
