# Supplementary material for: Synthesis, Internalization and Visualization of N-(4-Carbomethoxy) Pyrrolidone Terminated PAMAM [G5:G3-TREN] Tecto(dendrimers) in Mammalian Cells
Source: Molecules. 2020 Sep 25;25(19):4406. doi: 10.3390/molecules25194406 (PMC7583011; doi:10.3390/molecules25194406)
Supplement: Supplementary file 1 [file molecules-25-04406-s001.pdf]

Supplementary Materials for

Synthesis, Internalization and Visualization of *N*-(4-Carbomethoxy) Pyrrolidone Terminated PAMAM [G5:G3-TREN] Tecto(dendrimers) in Mammalian Cells

Maciej Studzian [1,2], Paula Działak [1], Łukasz Pułaski [2,3], David M. Hedstrand [4], Donald A. Tomalia [4,5,6,*] and Barbara Klajnert-Maculewicz [1,7,*]

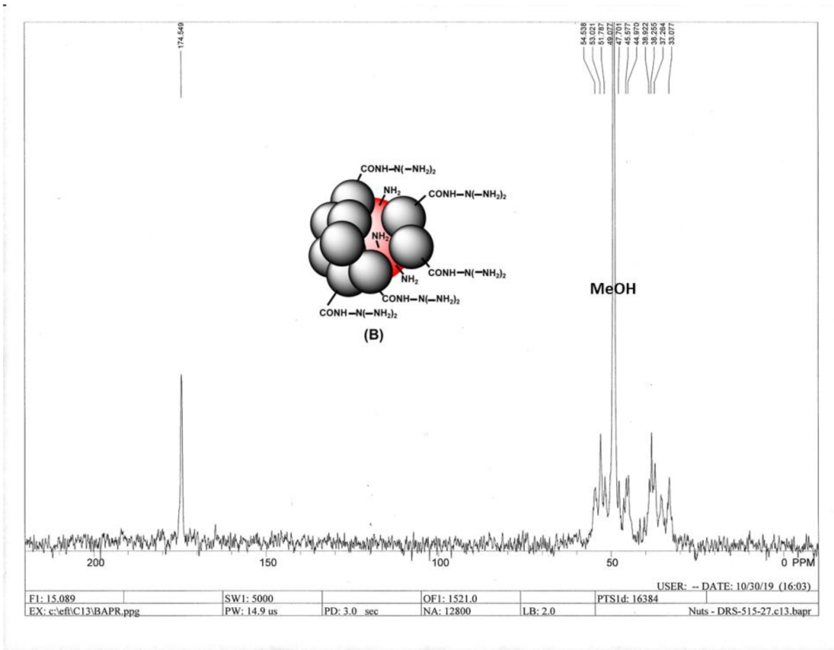

Figure S1. ^{13}C-NMR spectra of Core-Shell tecto(dendrimer) Structure (B).

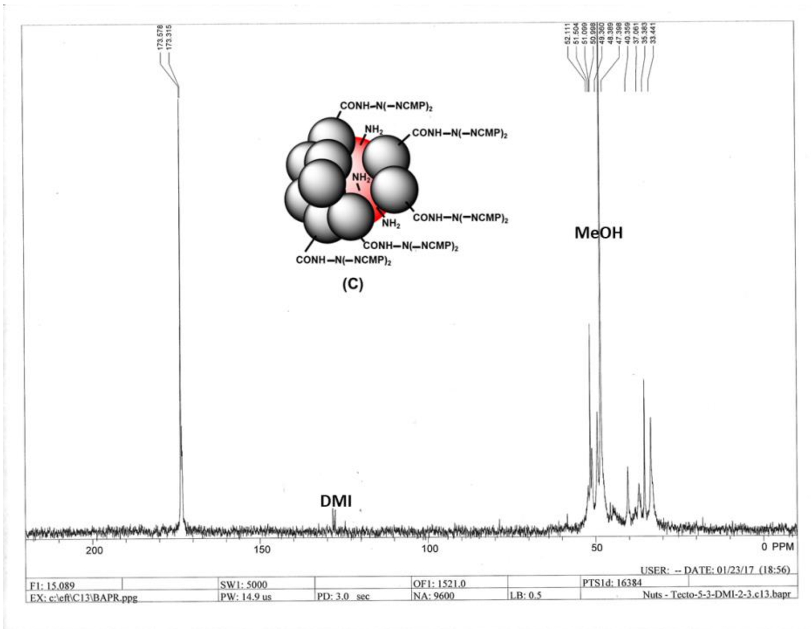

Figure S2. ^{13}C-NMR spectra of Core-Shell tecto(dendrimer) Structure (C).

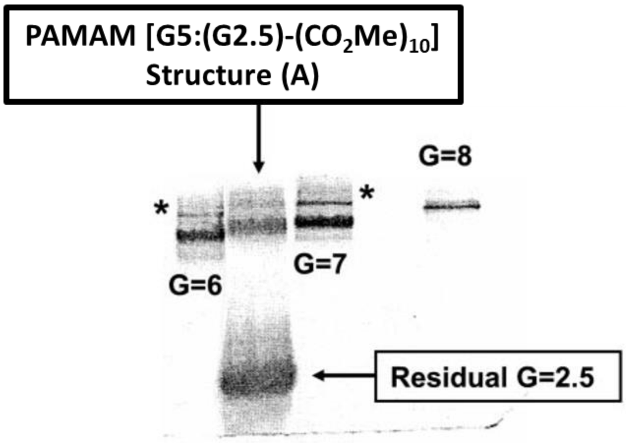

Figure S3. PAGE analysis of PAMAM [G5:(G2.5)-$(CO_2Me)_{10}$] , Structure (A) prepared according to Route I as described in (DATomalia et.al. PNAS,(2002),99, 8, 5081-5087). This product is compared with PAMAM dendrimer tectons (i.e., G=6,7,8) containing their respective dimers (*)

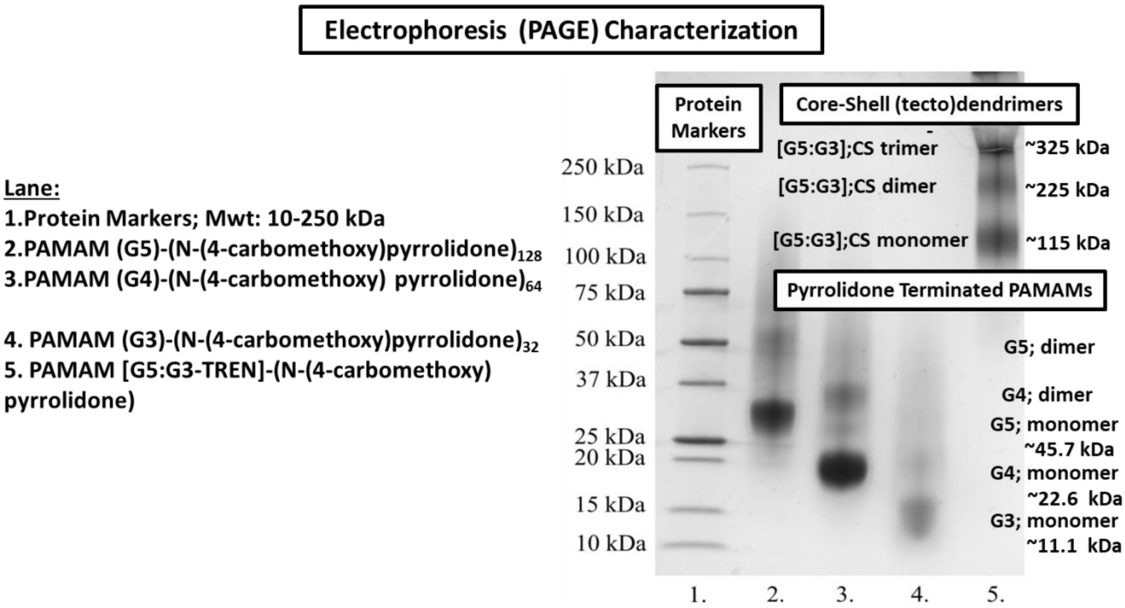

Figure S4. PAGE of Core-Shell PAMAM,[G5:G3-TREN]-N-(4-Carbomethoxy) pyrrolidone terminated, Structure (C) compared with protein markers and monomeric PAMAM (G3-5)-N-(4-Carbomethoxy) pyrrolidone terminated, dendrimers.
